# Interfacial Charge Transfer in MoS_2_/TiO_2_ Heterostructured Photocatalysts: The Impact of Crystal Facets and Defects

**DOI:** 10.3390/molecules24091769

**Published:** 2019-05-07

**Authors:** Tingcha Wei, Woon Ming Lau, Xiaoqiang An, Xuelian Yu

**Affiliations:** 1 Beijing Computational Science Research Center, Beijing 100193, China; wtc@csrc.ac.cn; 2 Center for Green Innovation, School of Mathematics and Physics, University of Science & Technology Beijing, Beijing 100083, China; 3 Center for Water and Ecology, State Key Joint Laboratory of Environment Simulation and Pollution Control, School of Environment, Tsinghua University, Beijing 100084, China; 4 Beijing Key Laboratory of Materials Utilization of Nonmetallic Minerals and Solid Wastes, National Laboratory of Mineral Materials, School of Materials Science and Technology, China University of Geosciences, Beijing 100083, China; xlyu@cugb.edu.cn

**Keywords:** crystal facets, oxygen vacancy, molybdenum sulfide, titanium dioxide, photocatalysts, charge transfer

## Abstract

One of the most challenging issues in photocatalytic hydrogen evolution is to efficiently separate photocharge carriers. Although MoS_2_ loading could effectively improve the photoactivity of TiO_2_, a fundamental understanding of the charge transfer process between TiO_2_ and MoS_2_ is still lacking. Herein, TiO_2_ photocatalysts with different exposed facets were used to construct MoS_2_/TiO_2_ heterostructures. XPS, ESR, together with PL measurements evidenced the Type II electron transfer from MoS_2_ to {001}-TiO_2_. Differently, electron-rich characteristic of {101}-faceted TiO_2_ were beneficial for the direct Z-scheme recombination of electrons in TiO_2_ with holes in MoS_2_. This synergetic effect between facet engineering and oxygen vacancies resulted in more than one order of magnitude enhanced hydrogen evolution rate. This finding revealed the elevating mechanism of constructing high-performance MoS_2_/TiO_2_ heterojunction based on facet and defect engineering.

## 1. Introduction

Photocatalysis based on semiconductor nanomaterials has been extensively studied during the past decades, due to its great potential to solving the worldwide energy and environmental crisis [1,2]. Due to the abundance and high chemical stability, TiO_2_ is one of the most widely used semiconductors for photocatalysis [3,4,5]. However, its large-scale application is seriously limited by its poor light absorption and the fast recombination of electron–hole pairs. Tremendous strategies have been used to improve the photoreactivity of TiO_2_, such as morphology control, microstructure modulation, co-catalyst loading and metal/nonmetal doping [6,7,8,9]. Among these strategies, heterojunction design has proved to be the most promising way to separate photo-induced charge carriers [10,11].

MoS_2_, a typical layered transition-metal dichalcogenide, attracted a lot of recent attention because of its narrow energy gap and large surface area [12,13,14]. Although a fundamental understanding of the heterostructured interface between TiO_2_ and MoS_2_ is still lacking, as-constructed junctions phenomenologically exhibited remarkably enhanced activity for photocatalytic hydrogen evolution. For example, several reports demonstrated that photo-generated electrons efficiently transferred from the conduction band of TiO_2_ to MoS_2_ [15,16,17,18,19]. The role of MoS_2_ as electron reservoir renders it a potential candidate to replace the costly noble metal cocatalysts. In contrast, several recent studies have revealed the Type II band alignment between TiO_2_ and MoS_2_. This meant that electrons moved from the conduction band of MoS_2_ to that of TiO_2_ [20,21,22]. This controversy was due to the fact that many factors can affect the charge transfer behavior around heterostructured interfaces. Up to now, the lithium-free fabrication of trigonal phase MoS_2_ with metallic characteristic remains challenging. Ordinarily, the tunable bandgap of hexagonal phase MoS_2_ (1.3–1.9 eV) unavoidably complicated the interfacial mechanism [16,23]. Moreover, the charge carrier behavior in semiconductor photocatalyst is strongly dependent on the exposed facets and structural defects. For example, {001} and {101} facets of TiO_2_, two most common facets of anatase phase, usually exhibit different adsorption characteristics and redox abilities during the photocatalytic reaction [24,25,26,27]. Our research further revealed the significant contribution of facet-dependent formation of oxygen vacancy defects on the interfacial behavior of photo-induced charge carriers [28]. In this regard, it is a critical point to investigate the impact of crystal facets and defects on the charge separation in MoS_2_-grafted faceted TiO_2_.

In this paper, {001} and {101}-faceted TiO_2_ were used to construct MoS_2_/TiO_2_ heterostrutures. The influence of exposed facets and oxygen vacancy defects on the interfacial separation of charge carriers was fundamentally investigated. Experimental characterizations indicated that {001} facets of TiO_2_ are apt to accept electrons from MoS_2_, while {101} facets are favorable for the Z-scheme recombination of electrons in TiO_2_ with holes in MoS_2_. The facet-dependent charge transfer process and the defect-enhanced charge separation pave a novel way to design high-efficiency heterostructured photocatalysts for energy and environmental applications.

## 2. Results and Discussion

Facile hydrothermal reaction was used to fabricate {001}-faceted (T001) and {101}-faceted TiO_2_ (T101). Then, as-synthesized TiO_2_ was heat treated under hydrogen atmosphere to obtain defective {001}-TiO_2_ (V_o_-T001) and {101}-TiO_2_ (V_o_-T101). Different types of TiO_2_ was hybridized with commercial MoS_2_ by ultrasonication (Figure S1). MoS_2_-grafted {001}- and {101}-faceted TiO_2_ without and with oxygen vacancies were denoted as T001/MoS_2_, V_o_-T001/MoS_2_, T101/MoS_2_ and V_o_-T101/MoS_2_, respectively.

**Figure 1:**
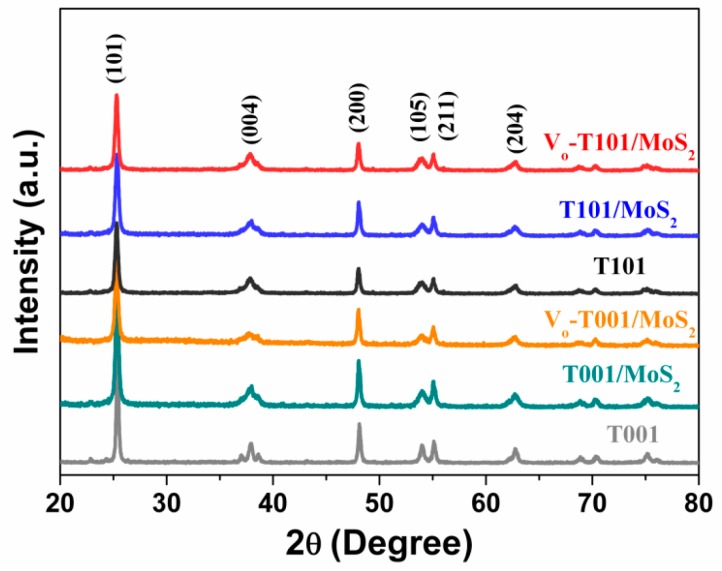
XRD patterns of perfect and defective faceted TiO_2_ before and after the deposition of MoS_2_.

The phase structure of as-prepared samples was examined by X-ray diffraction (XRD). As shown in Figure 1, all diffraction peaks can be indexed to anatase TiO_2_ (JCPDS card No. 01-078-2486). The diffraction peaks at 25.31° and 37.79° are ascribed to (101) and (004) planes, respectively [26,29]. Thermal reduction exhibits neglectable influence on the crystal phase and crystallinity of TiO_2_. No characteristic peaks of MoS_2_ are discernible in the composite photocatalysts due to the relatively lower amount of MoS_2_ and the high dispersity.

**Figure 2:**
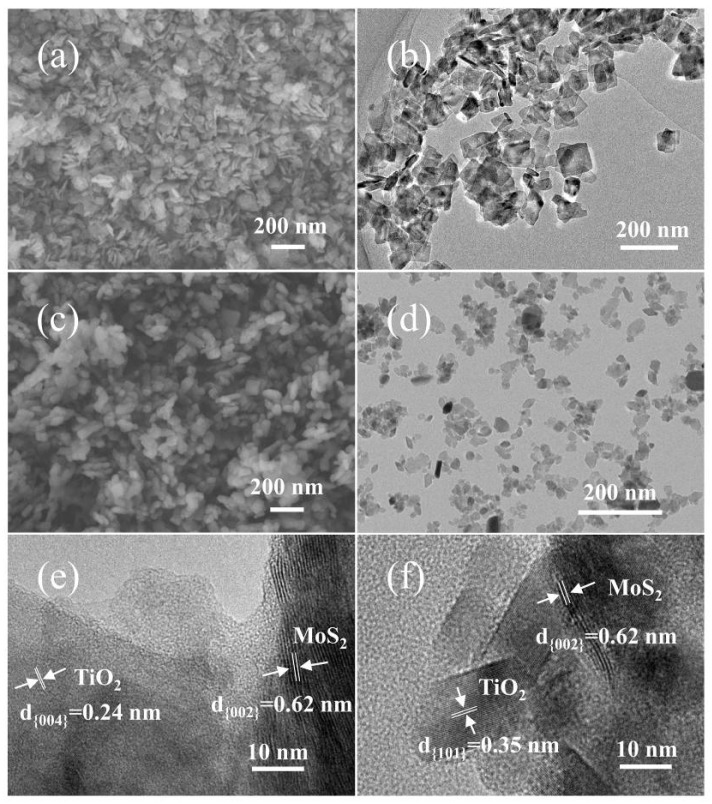
SEM image (**a**) and TEM image (**b**) of V_o_-T001/MoS_2_. SEM image (**c**) and TEM image (**d**) of V_o_-T101/MoS_2_. HR-TEM images of V_o_-T001/MoS_2_ (**e**) and V_o_-T101/MoS_2_ (**f**).

Figure 2 shows the morphology of Vo-T001/MoS_2_ and V_o_-T101/MoS_2_. Based on field emission scanning electron microscope (FE-SEM) and transmission electron microscope (TEM) observations, Vo-T001/MoS_2_ sample is composed of uniform square-shaped nanosheets with an average side size of 100 nm and thickness of ~10 nm (Figure 2a,b). V_o_-T101/MoS_2_ sample is observed for rhombic morphology with an average apex-to-apex diameter of ~15 nm (Figure 2c,d). Figure 2e presents the high-resolution TEM image of V_o_-T001/MoS_2_. The lattice spacing of 0.24 nm corresponds to the (004) plane of TiO_2_ [30]. Besides, the surface of square-shaped TiO_2_ is intimately coupled with layered nanosheets MoS_2_. The interlayer distance of 0.62 nm corresponds to the (002) planes of hexagonal MoS_2_. The formation of MoS_2_/{101}-faceted TiO_2_ can be further confirmed by Figure 2f.

**Figure 3:**
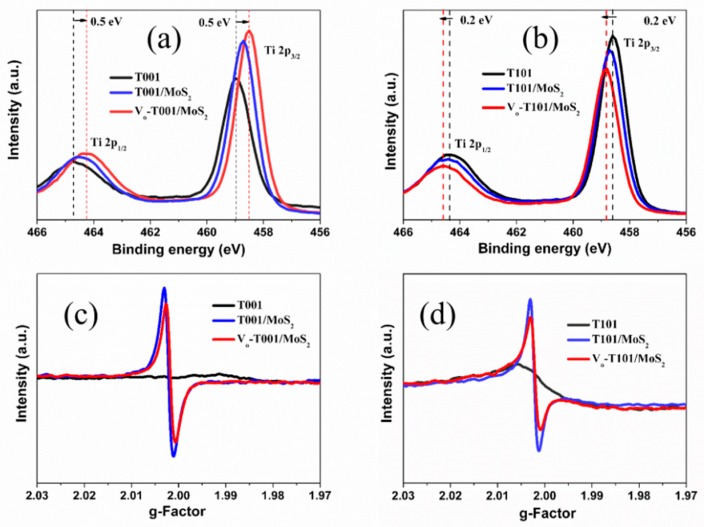
Ti 2p XPS spectra of {001}-faceted TiO_2_ (**a**) and {101}-faceted TiO_2_ (**b**) before and after the deposition of MoS_2_ with and without defect. ESR pattern of 001-faceted TiO_2_ (**c**) and 101-faceted TiO_2_ (**d**) before and after the deposition of MoS_2_ with and without defect.

X-ray photoelectron spectroscopy (XPS) was used to investigate the chemical states of component elements. Figure 3a exhibits Ti 2p XPS spectra of {001}-faceted TiO_2_, MoS_2_/TiO_2_ heterostructures with and without oxygen vacancies. The Ti 2p peaks shift to lower binding energies after the deposition of MoS_2_ onto TiO_2_ nanosheets. It means that Ti atoms accept electrons from MoS_2_, resulting in more Ti^3+^ in the heterostructures. The creation of oxygen vacancies in TiO_2_ further accelerates this process, as the shift value increased to 0.5 eV for V_o_-T001/MoS_2_. The coupling of MoS_2_ with {101}-faced TiO_2_ results in totally different change of spectra. The shift of Ti 2p peaks to higher binding energies indicates that Ti atom was electron donor. The facet-dependent interfacial electronic structure implies the different charge transfer behavior in T001/MoS_2_ and T101/MoS_2_ heterostrutures.

The electronic structure of different samples was further studied by electron spin resonance (ESR). In Figure 3c, pristine {001}-faceted TiO_2_ presents a weak signal around g value of 1.98–1.99, which can be ascribed to electrons trapped by Ti^3+^ [30,31]. Differently, {101}-faceted TiO_2_ possesses a much stronger signal at 2.002 (Figure 3d). It indicates that the exposure of {101} facets is more favorable for the formation of surface oxygen vacancies [32,33]. The deposition of MoS_2_ onto T001 and T101 results in the significantly increased ESR signals. It means that there are strong interfacial interactions between MoS_2_ and TiO_2_. The retention of this signal in V_o_-T001/MoS_2_ and V_o_-T101/MoS_2_ proves the formation of defective heterostructured photocatalysts.

**Figure 4:**
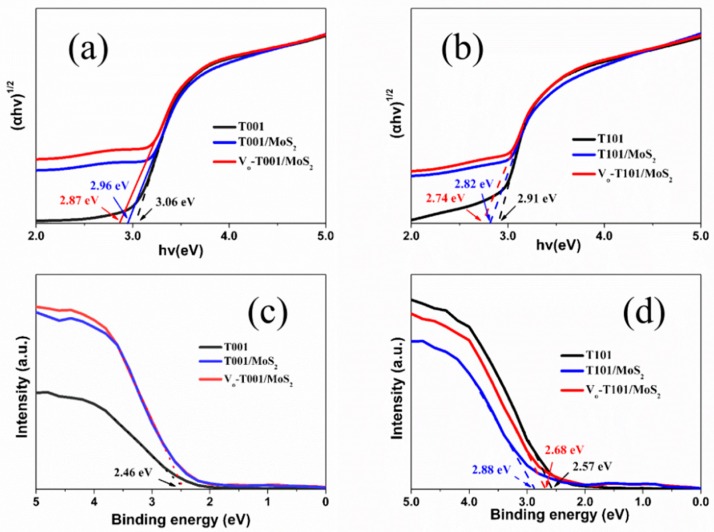
Tauc plots of {001}-faceted TiO_2_ (**a**) and {101}-faceted TiO_2_ (**b**) before and after the deposition of MoS_2_ with and without defect. VB XPS of {001}-faceted TiO_2_ (**c**) and {101}-faceted TiO_2_ (**d**) before and after the deposition of MoS_2_ with and without defects.

To evaluate the influence of MoS_2_ loading and defect formation on the light absorption ability of photocatalysts, UV-vis diffuse reflectance spectra were collected. As shown in Figure S3, {001}- and {101}-faceted TiO_2_ only present strong absorption in the UV light region. The formation of structural defects and subsequent loading of MoS_2_ result in the obvious visible light absorption. On the basis of Kubelka-Munk function, shown in Figure 4a,b, the corresponding band gaps of T001 and T101 are determined to be 3.06 and 2.91 eV, respectively.

In comparison, the band gap of V_o_-T001/MoS_2_ and V_o_-T101/MoS_2_ heterostructures are determined to be 2.87 and 2.74 eV, respectively. The influence of junction formation on the energy levels was investigated by valance band XPS. As shown in Figure 4c, all T001-based samples exhibit similar valence band positions, i.e. the negligible change of electronic structure. In contrast, the coupling of T101 with MoS_2_ leads to the 0.31 eV down-shift of valence band, while the shift value decrease to 0.11 eV after the introduction of oxygen vacancies in the heterostructures (Figure 4d). All these results demonstrate the facet-dependent electronic structure in MoS_2_/TiO_2_ heterostructures, which should exhibit impact on the behavior of interfacial charge separation.

**Figure 5:**
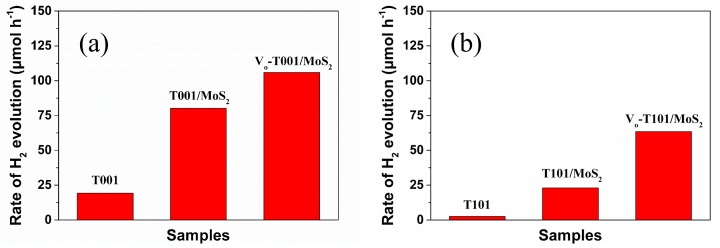
Photocatalytic activity of H_2_ evolution under UV light irradiation of (**a**) {001}-faceted TiO_2_ before and after the deposition of MoS_2_ with and without defect and (**b**) {101}-faceted TiO_2_ before and after the deposition of MoS_2_ with and without defect.

Photoactivity of different photocatalysts was thereafter evaluated by hydrogen evolution reactions. As show in Figure 5a, {001}-faceted TiO_2_ exhibits moderate activity, with a hydrogen evolution rate of 20 μmol h^−1^. After the deposition of MoS_2_, four-fold increased photocatalytic performance is achieved. An additional 30% enhancement is achieved after the creation of oxygen vacancies in {001}-TiO_2_. In comparison, poor photoactivity is achieved for {101}-faced TiO_2_. The formation of T101/MoS_2_ improves the hydrogen evolution rate from 3 μmol h^−1^ to 22 μmol h^−1^. The formation of defective structure presents significant impact on the photocatalytic performance. Vo-T101/MoS_2_ possesses the highest hydrogen production rate of 61 μmol h^−1^, which is about 20 and 3 times higher than pristine TiO_2_ and MoS_2_/TiO_2_ heterojunction. The above results indicate that the construction of MoS_2_/TiO_2_ heterostructures can efficiently improve the photocatalytic performance of TiO_2_. Moreover, defect modulation is a more effective strategy to improve the photoactivity of {101}-faceted TiO_2_.

In order to study the synergetic effect between oxygen vacancy defects and MoS_2_, the charge carrier behavior was studied by photoluminescence (PL) measurements. Obviously, the deposition of MoS_2_ and oxygen vacancy formation results in the different change of fluorescence emission of {001}- and {101}-faceted TiO_2_.

**Figure 6:**
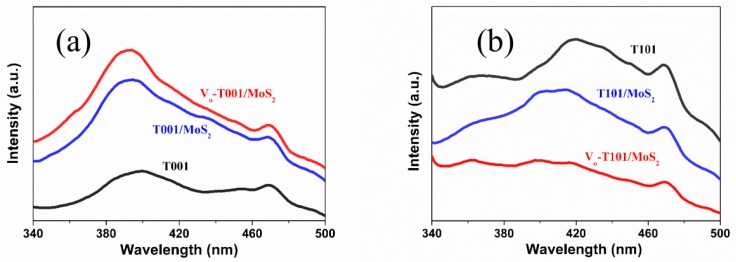
PL emission spectra of {001}-faceted TiO_2_ (**a**) and {101}-faceted TiO_2_ (**b**) before and after the deposition of MoS_2_ with and without defect.

In Figure 6a, the transition emission of T001/MoS_2_ is much higher than pristine TiO_2_, implying the possible electron transfer from MoS_2_ to TiO_2_. Differently, the formation of heterostructure and defective interface leads to the obvious PL quenching of {101}-faceted TiO_2_ (Figure 6b) [21,34,35]. The improved separation of charge carriers can be further evidenced by the reduced radius of semi-circle in the Nyquist plots in Figure S2.

**Figure 7:**
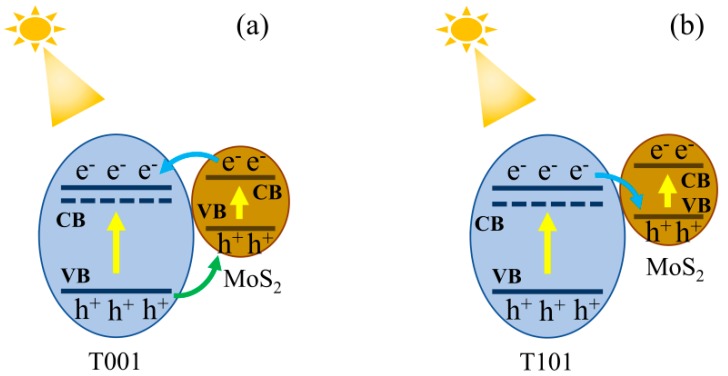
Scheme representation of charge separation in heterojunction for (**a**) V_o_-T001/MoS_2_ and (**b**) V_o_-T101/MoS_2_.

Based on the above results, the charge transfer mechanism in MoS_2_-grafted faceted TiO_2_ is illustrated in Figure 7a,b. Firstly, according to the previous reports, the purchased commercial MoS_2_ was readily 2H-MoS_2_ with a hexagonal structure [36,37]. Theoretically, this type MoS_2_ in the faceted heterostructures should not be simply considered as co-catalysts for hydrogen generation. The different coordination environment of component atoms in {001}- and {101}-faceted TiO_2_ further complicate this case. It should be noted that the amount of 5-fold-coodinated Ti on {001} facets (100%) is much larger than {101} facets (50%) of TiO_2_ [38]. With the creation of oxygen vacancies, undercoodinated Ti4c on the surface of {001} facets can expectedly provide sufficient reactive sites for photocatalysis. However, defect modulation seemed to be more effective to improve the performance of T101/MoS_2_ heterostructures. It indicated that this improvement was resulted from the interfacial process of charge carriers, rather than intrinsic electronic structures. Based on the UV and valence-bond XPS measurements, both the conduction band and valence band of {101}-TiO_2_ are more positive than {001}-TiO_2_. Therefore, more electrons should migrate to the {101} facets of TiO_2_, while holes accumulate on the {001} facets. It means that {001}-TiO_2_ are prone to accept electrons from MoS_2_, i.e., the formation of Type II junction between MoS_2_ and {001}-TiO_2_. As shown in Figure 7a, this charge transfer process is further facilitated by the construction of defective structure. However, the deposition of MoS_2_ onto the electron-rich {101}-TiO_2_ results in remarkable down-shift of band gap energy levels. Its much lower conduction band is favorable for the direct recombination of these electrons with holes in the valence band of MoS_2_, analogous to the Z-scheme mechanism [39]. When oxygen vacancies are introduced into this system, the mid-gap defect states can act as electron mediator to facilitate this charge transfer. The synergetic effect between oxygen vacancies and crystal facets leads to the more than an order of magnitude increase in hydrogen production activity.

## 3. Materials and Methods

### 3.1. Fabrication of {001}-Faceted TiO_2_

To fabricate {001}-faceted TiO_2_ (T001), 1.5 mL of hydrofluoric acid was added into 12.5 mL of titanium (IV) butoxide under vigorous stirring. After 30 min, the solution was transferred into a Teflon autoclave with a capacity of 50 mL. A hydrothermal reaction was carried out at 200 °C for 24 h. When the autoclave was cooled to room temperature, the precipitants were separated by high-speed centrifugation. To remove the residual fluorine ions, the powders were soaked in 0.1 M NaOH solution for 12 h. After the fully rinsing and drying, white-colored TiO_2_ powders were obtained, which were annealed at 400 °C for 2 h in a muffle furnace.

### 3.2. Fabrication of {101}-Faceted TiO_2_

A two-step hydrothermal method was used to fabricate TiO_2_ with exposed {101} facets (T101) [40]. In a typical procedure, 1 g of P25 TiO_2_ nanoparticles was hydrothermally treated with 50 mL 17 M of KOH solution in a Teflon autoclave at 110 °C for 24 h. The resulting precipitates were washed and neutralized using DI water and acetic acid aqueous solution, respectively. Then the powders dried in oven for 12 h. The dried titanate powders 750 mg were further dispersed into 15 mL of ultrapure water under strong stirring. Another hydrothermal reaction was carried out at 170 °C for 24 h. Finally, the white TiO_2_ nanoparticles were centrifuged and dried overnight. Then the powders were annealing at 400 °C for 2 h in a muffle furnace.

### 3.3. Synthesis of Nonstoichiometric TiO_2_ Nanostructures

A thermal reduction strategy was used to fabricate defective TiO_2_. Typically, as-synthesized TiO_2_ were placed in the middle of a horizontal tube furnace. The powders were heat treated at 400 °C for 2 h in a H_2_/Ar flow (20 mL/min).

### 3.4. Fabrication of MoS_2_/TiO_2_ Heterostructures

**Scheme 1:**
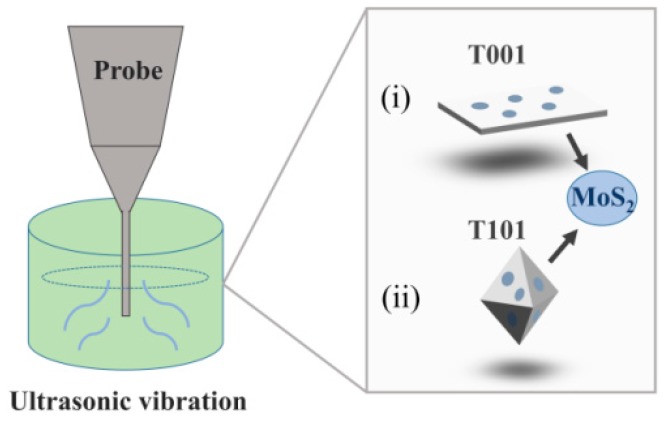
Fabrication of MoS_2_/TiO_2_ heterostructures.

The fabrication procedures of MoS_2_/TiO_2_ heterostructure were illustrated in Scheme 1. Typically, commercial MoS_2_ (Nanjing XFNANO Materials Tech Co., Ltd, Nanjing, China) were added into deionized water and sonicated for 8 h using ultrasonic cell disruptor (Biosafer 900-92, Nanjing Safer Biotech Co., Ltd., Nanjing, China). Then, 100 mg TiO_2_ and a certain amount of MoS_2_ dispersion were mixed in 50 mL of deionized water. After one hour’s ultrasonication and two hours’ stirring, the precipitation was filtrated, washed and dried to achieve MoS_2_/TiO_2_ composites. The weight ratio of MoS_2_ in the composites was kept at 1%. All samples were prepared with the same method, using TiO_2_ with different exposed facets and electronic structures. MoS_2_-grafted {001}- and {101}-faceted TiO_2_ without and with oxygen vacancies were denoted as T001/MoS_2_, V_o_-T001/MoS_2_, T101/MoS_2_ and Vo-T101/MoS_2_, respectively.

### 3.5. Characterizations

X-ray diffraction (XRD) was carried out on X’Pert Pro MPD (PANalytical B.V., Almelo, the Netherlands). X-ray photoelectron spectroscopy (XPS) and valence band was performed on an ESCALAB MKII spectrometer (VG Scientific Ltd., London, UK) with an Mg Kα excitation source. The morphology of catalysts were observed by field emission scanning electron microscope (FE-SEM, SIGMA, Jena, Germany) and high-resolution transmission electron microscope (HR-TEM, JEOL-2100F, JEOL, Tokyo, Japan). UV-vis diffusion reflectance spectra (DRS) were acquired on an UV−vis−NIR spectrophotometer (Cary 5000, Varian, Palo Alto, CA, USA). The steady-state fluorescence and the time-resolved fluorescence spectra were measured on a fluorescence spectrometer (FLS-980, Edinburgh Instruments Ltd., Edinburgh, UK). Electron spin resonance (ESR) analysis was carried out using a Bruker E500 spectrometer (Bruker BioSpin Gmbh, Karlsruhe, Germany).

### 3.6. Photocatalytic Experiments

Photocatalytic hydrogen evolution reaction was carried out in a closed gas-circulation system. Typically, 15 mg of different photocatalysts were dispersed into 100 mL aqueous solution containing 10 mL of methanol as hole scavenger. A 300 W Xe lamp (CEL-HXF300, Ceaulight, Beijing, China) was used as the light source. The evoluted H_2_ was analyzed by an online gas chromatograph equipped with a column of 5 Å molecular sieves (GC-7806, Shiweipuxin, Beijing, China).

### 3.7. Electrochemical Measurements

The photoelectrochemical properties were investigated in a three-electrode cell using an electrochemical workstation (CHI800D, CH Instruments, Shanghai, China). The catalyst loaded FTO glass, Pt wire and Ag/AgCl electrode were used as the working electrode, counter electrode and reference electrode, respectively. 0.5 M Na_2_SO_4_ solution was used as electrolyte. The electrical impedance spectroscopy (EIS) was measured at an applied potential of 0 V vs. Ag/AgCl. The Mott-Schottky curves were collected at the frequency of 1000 Hz.

## 4. Conclusions

In summary, we have revealed that the mechanism of interfacial charge transfer in MoS_2_/TiO_2_ heterostructures was highly dependent with the exposed facets of TiO_2_. Due to the electron-rich characteristic and the subsequent band alignment, {101} facets of TiO_2_ were more favorable for the direct Z-scheme charge transfer. The introduction of oxygen vacancies into Type II heterojunction resulted in the 5-fold increased hydrogen evolution rate over MoS_2_/{001}-TiO_2_, while more than one order of magnitude increased activity was achieved for Z-scheme MoS_2_/{101}-TiO_2_. Our strategy presents a paradigm for the rational design of heterostructured photocatalysts with controlled charge transfer pathways toward photocatalytic solar energy conversion.

## Supplementary Materials

The following are available online: Figure S1: TEM image of commercial MoS_2_ nanosheets, Figure S2: UV-vis diffuse reflectance spectra of {001}-faceted TiO_2_ (a) and {101}-faceted TiO_2_ (b) before and after the deposition of MoS_2_ with and without defect, Figure S3: EIS plots of {001}-faceted TiO_2_ before and after the deposition of MoS_2_ with and without defect.
