# Supplementary material for: Interfacial Charge Transfer in MoS2/TiO2 Heterostructured Photocatalysts: The Impact of Crystal Facets and Defects
Source: Molecules. 2019 May 7;24(9):1769. doi: 10.3390/molecules24091769 (PMC6539887; doi:10.3390/molecules24091769)
Supplement: Supplementary file 1 [file molecules-24-01769-s001.pdf]

molecules

MDPI

Article

Interfacial Charge Transfer in MoS_2/TiO_2 Heterostructured Photocatalysts: the Impact of Crystal Facets and Defects

Tingcha Wei [1], Woon Ming Lau [2,*], Xiaoqiang An [3,*] and Xuelian Yu [4]

[1] Beijing Computational Science Research Center, Beijing 100193, China; wtc@csrc.ac.cn
[2] Center for Green Innovation, School of Mathematics and Physics, University of Science & Technology Beijing, Beijing 100083, China.
[3] Center for Water and Ecology, State Key Joint Laboratory of Environment Simulation and Pollution Control, School of Environment, Tsinghua University, Beijing 100084, China.
[4] Beijing Key Laboratory of Materials Utilization of Nonmetallic Minerals and Solid Wastes, National Laboratory of Mineral Materials, School of Materials Science and Technology, China University of Geosciences, Beijing 100083, China; xlyu@cugb.edu.cn
* Correspondence: leolau@ustb.edu.cn (W.M.L.); xqan@tsinghua.edu.cn (X.A.)

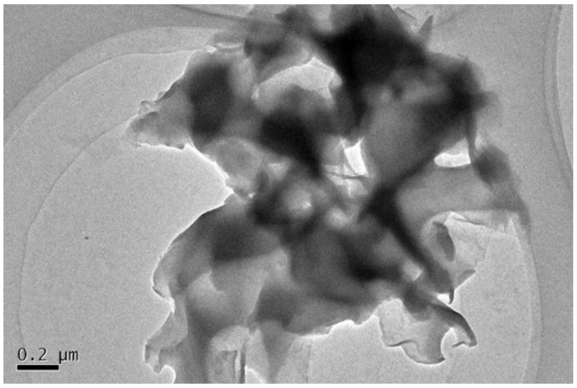

Fig. S1. TEM image of commercial MoS_2 nanosheets.

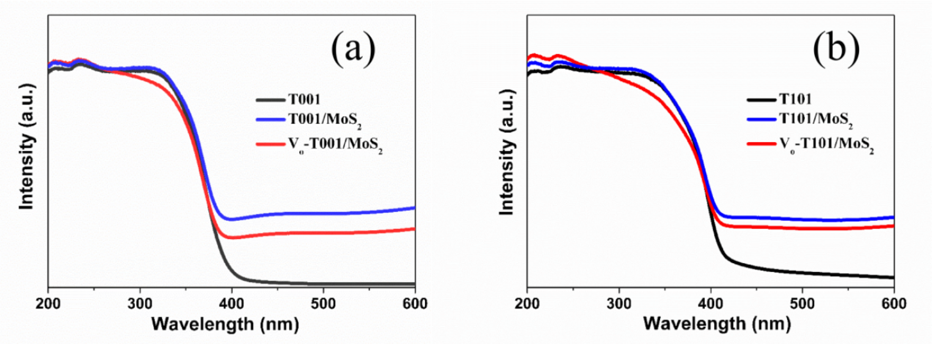

Fig. S2. UV-vis diffuse reflectance spectra of {001}-faceted TiO_2 (a) and {101}-faceted TiO_2 (b) before and after the deposition of MoS_2 with and without defect.

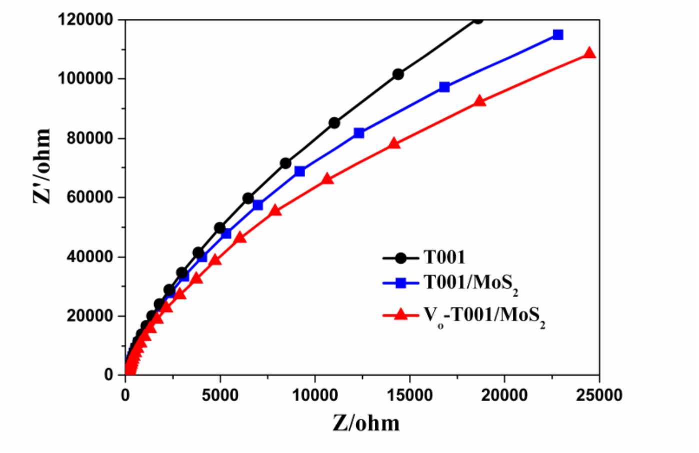

Fig. S3. EIS plots of {001}-faceted TiO_2 before and after the deposition of MoS_2 with and without defect.
